# A simple and inexpensive method to monitor and minimize exposure from manipulation of cytotoxic drugs

**DOI:** 10.1177/10781552231173878

**Published:** 2023-05-07

**Authors:** Nuro Ali, Helena Carmo, Raquel Robalo, Luísa Rocha, Cristina Fernandes, Fernando Moreira

**Affiliations:** 1 Associate Laboratory i4HB - Institute for Health and Bioeconomy, Department of Biological Sciences, Laboratory of Toxicology, Faculty of Pharmacy, University of Porto, Porto, Portugal; 2 UCIBIO/REQUIMTE, Department of Biological Sciences, Laboratory of Toxicology, Faculty of Pharmacy, University of Porto, Porto, Portugal; 3 Serviço Farmacêutico do Centro Hospitalar de Vila Nova de Gaia/Espinho, E.P.E., Vila Nova de Gaia, Portugal; 4 Escola Superior de Saúde, Instituto Politécnico do Porto, Rua Dr António Bernardino de Almeida, Porto, Portugal; 5 Centro de Investigação em Saúde e Ambiente, Escola Superior de Saúde, Instituto Politécnico do Porto, Rua Dr António Bernardino de Almeida, Porto, Portugal

**Keywords:** Cytotoxic drugs, antineoplastic, hazardous drugs, occupational exposure

## Abstract

Pharmacy personnel that manipulate cytotoxic drugs are under continuous exposure risk. Therefore, training and strict adherence to recommended practices should always be promoted. The main objective of this study was to develop and apply a safe, effective and low-cost method for the training and assessment of the safe handling of cytotoxic drugs, using commercially available tonic water. To evaluate the potential of tonic water as a replacement marker for quinine hydrochloride, deliberate spills of 1 mL of four different tonic waters (one coloured and three non-coloured) were analysed under ultraviolet light (300–400 nm). The pigmented sample did not produce fluorescence under ultraviolet (UV) light. The three commercially available tonic waters that exhibited fluorescence were further analysed by UV/Vis spectrophotometry (300–500 nm). Afterwards, a protocol of simulated manipulation of cytotoxic drugs was developed and applied to 12 pharmacy technicians, that prepared 24 intravenous bags according to recommended routine procedures using tonic water. Participants responded to a brief questionnaire to evaluate the adequacy and applicability of the activity. Seven of the participants had spillages during manipulation, the majority of which recorded during manipulation with needles. All participants scored the tonic water manipulation simulation with 4 or 5 points for simplicity, efficiency and feasibility. The obtained results suggest that tonic water can be used to simulate the manipulation of cytotoxic drugs in training and assessment programs. By using this replacement marker for quinine hydrochloride, it is possible to perform a more cost-effective, yet equally effective, assessment.

## Introduction

One of the main hurdles for the safe handling of cytotoxic drugs is the poor investment in the skills and knowledge of the professionals involved in these tasks. Adequate training increases knowledge and contributes to the safe handling of dangerous drugs by adopting exposure control measures.^1^ There is abundant evidence of the detrimental health effects related to cytotoxic drug handling. Previous studies showed statistically higher DNA damage in lymphocytes of nurses exposed to cytotoxic drugs than in non-exposed controls.^2,4^ The prevalence of micronuclei (DNA fragments) in epithelial cells and peripheral lymphocytes of healthcare workers exposed to cytotoxic drugs was also found to be statistically higher compared to controls.^3,5^ There is also evidence to suggest that oxidative stress is increased in exposed professionals.^6^

The International Society of Oncology Pharmacy Practitioners,^7^ the Occupational Safety and Health Administration (OSHA)^8^ and the American Society of Health-System Pharmacists^9^ recommend that employers mandatorily ensure training before starting handling these drugs. Additionally, once enrolled in cytotoxic drug manipulation, pharmacy professionals should receive continuous training and be submitted to assessment, at least, yearly.^7^ Practical training and assessment can be done using stained solutions or solutions that exhibit fluorescence under ultraviolet (UV) light (such as quinine hydrochloride).^10^ These trainings and assessments enable the recognition of the possible location of contamination during operators’ handling and, if necessary, to establish corrective measures aimed at preserving the environment and the safety of professionals.^10,11^

The present study aimed at developing and implementing a low-cost method to efficiently monitor spills, during training and assessment programs of pharmacy professionals that manipulate cytotoxic drugs.

## Methodology

### Adaptation and application of the quinine test using tonic water

#### Fluorescence and spectrophotometric analysis after deliberate tonic water spills

Four commercially available tonic waters – three non-coloured tonic waters and one coloured tonic water – were deliberately spilled (1 mL) in working fields for cytotoxic handling (Chemoprotect^®^), and the produced fluorescence was monitored under 300–400 nm UV light (BeamZ^®^) (Table 1).

**Table 1.** Description of the commercial tonic waters included in the present study.

Sample	Brand	Ingredients	Native colouration
A	1	Water; sugar; carbon dioxide; acidifier: citric acid; natural flavours; quinine flavouring; sweeteners: acesulfame k and sucralose.	Non-coloured
B	1	Water; sugar; carbon dioxide; natural flavours; acidifier: citric acid; colour: anthocyanins; sweetener: steviol glycosides; carrot and safflower concentrate; quinine flavouring.	Coloured
C	2	Carbonated water; glucose and fructose syrup; acidifier: citric acid; preservative: sodium benzoate; flavouring: quinine.	Non-coloured
D	3	Carbonated water; glucose–fructose syrup; sugar; acidifier citric acid; preservative: potassium sorbate; flavourings (including quinine); sweetener (sucralose).	Non-coloured

An ultraviolet/visible (UV/Vis) spectrophotometer from VWR^®^ (UV-3100PC) was used to perform the UV/Vis spectrophotometric analysis of the tonic water samples that exhibited fluorescence in quartz cuvettes (VWR^®^). All tonic water samples were previously submitted to a degassing procedure in an ultrasonic cleaner (Sonorex^®^) for 15 min, at room temperature.

The UV/Vis spectrophotometric analysis was also performed for two quinine solutions (concentrations of 0.05 mol/L and 0.1 mol/L). Quinine solutions were prepared by dilution of quinine hydrochloride solution (250 mg/mL) (Labesfal^®^, Portugal). Determinations were performed in triplicate, for 11 different wavelengths, at intervals of 20 nm, between 300 nm and 500 nm.

#### Evaluation of manipulation practices using tonic water

To assess the handling procedures of professionals with prior experience in cytotoxic drugs’ manipulation, the presence of fluorescence traces after simulated manipulation with tonic water was analysed, using 300–400 nm UV light (BeamZ^®^).

Twelve pharmacy technicians compounded a total of 24 pockets (250 mL empty infusion bags from Hospira^®^) with tonic water (two infusion bags per operator). The manipulations were performed with 30 mL syringes (B Braun^®^) and included aspiration procedures of the vial containing tonic water and compounding procedures in the infusion bag, through Cyto-Set liquid transfer systems (B Braun^®^). The surface where simulated manipulation occurred was covered by an appropriate working field for cytotoxic handling (Chemoprotect^®^), and sterile gauze dressings (Bastos e Viegas^®^) were used in all connections between devices in which spills of tonic water could occur (syringe-needle; syringe-spike; syringe-Cyto-Set transfer system). For comparison purposes, when performing solution aspiration, half of the preparations were performed with a spike (Mini-spike, B Braun^®^), and half of the preparations were performed with a needle (19G, BD^®^). The protocol of simulated manipulation was based on routine manipulations in daily practice. Participants were informed orally and in writing of the objectives of the activity as well as the procedure protocol and freely consented in participating. A total of 288 surfaces of materials and equipment were evaluated during and after manipulation.

#### Questionnaire to participants that performed the manipulation with tonic water

A brief questionnaire, composed of seven items was presented to pharmacy technicians, after their participation in the manipulation with tonic water. The questionnaire was reviewed by two experts in the field of cytotoxic drug handling concerning the relevance of all questions, to ensure that any questions beyond the scope of the research were excluded. Providing succinct questionnaires is associated with increased participants’ adherence.^12^

All questions presented 5-point Likert scales, containing 1–5 answer options – 1 = strongly disagree, 2 = disagree, 3 = neither agree or disagree, 4 = agree and 5 = strongly agree. Likert scales provide a more reliable measure of strength for attitudes or beliefs than ambiguous terms.^12^

The questions focused the simplicity, efficiency, feasibility (related to the accessibility of the materials needed to perform the simulation), similarity to routine practices, adequacy to an assessment of pre-admission on cytotoxic drugs manipulation; adequacy to regular post-admission assessment on cytotoxic drugs manipulation; and adequacy to recognition of the areas/materials most susceptible to contamination.

Informed consent was provided by all participants and anonymity of responses was guaranteed. The study was approved by the Ethics Committee of the Centro Hospitalar de Vila Nova de Gaia/Espinho, E.P.E. (Document approved no. 79/2021).

## Results and discussion

After the deliberate spillages of non-coloured tonic water from three different commercially available brands, it was possible to easily visualize the fluorescence exhibited by spills, under UV light. Despite being from the same brand as one of the non-coloured samples, the coloured tonic water (with anthocyanins in the composition) did not exhibit fluorescence, under the same conditions.

**Figure 1.**
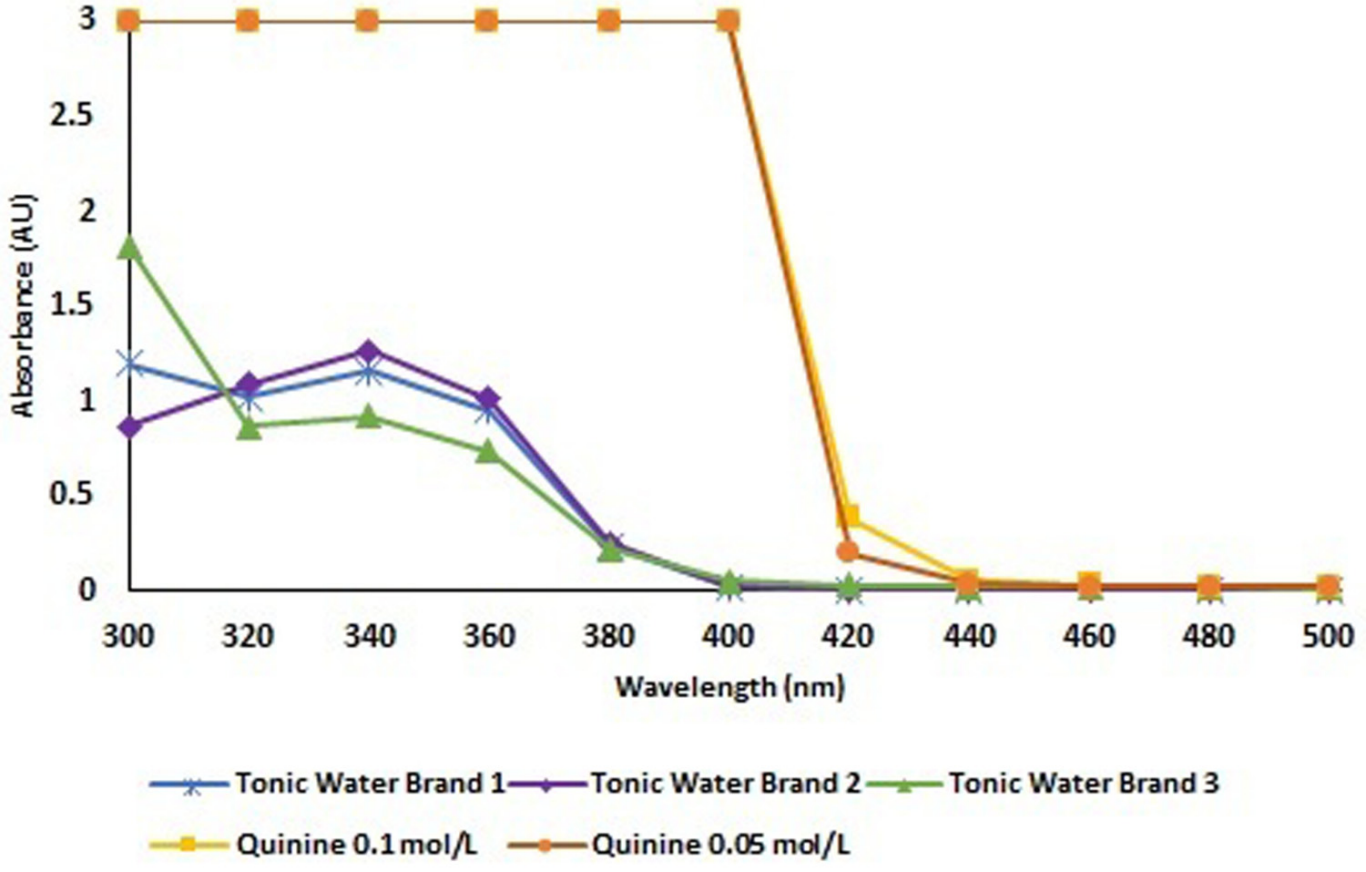
Absorbance of quinine solutions (0.005 mol/L and 0.1 mol/L) and three commercially available tonic waters, determined by ultraviolet/visible (UV-Vis) spectrophotometry.

Using commercially available and inexpensive tonic water, we have successfully detected the occurrence of spills during the simulated manipulation. Previous studies on training and evaluation with quinine have simulated the aspiration of 2 mL of a 0.1 mol/L quinine solution and transfer to an infusion bag, either containing 0.9% sodium chloride^10^ or 5% glucose,^11^ resulting in a final concentration of 0.04 mol/L. We have also confirmed that spillages could be recognized with quinine at a wavelength between 300 nm and 420 nm (Figure 1). It was also possible to confirm the detection of the absorbance exhibited by three different brands of tonic water, between 300 nm and 400 nm. However, despite the relative homogeneity between the different tested tonic waters, the corresponding absorbance values were lower than those recorded with quinine solutions at concentrations of 0.1 mol/L and 0.05 mol/L (Figure 1).

Since the analytical standard of quinine hydrochloride, of high purity, is not always immediately accessible and has a considerable cost, tonic water can thus be considered an interesting alternative. This alternative might be particularly relevant for less-resourced settings, where non-adherence to some preventive exposure measures is described.^13^ Despite the spectrophotometric results suggesting that the detection of spillages might be more sensitive with prepared quinine solutions than with tonic water, the visual inspection of deliberate spills of quinine solution 0.05 mg/mL (1 mL) or tonic waters (1 mL; Brands 1–3) revealed identical results with the naked eye, after illumination with UV light, and clearly distinct from the sodium chloride 0.9% control solution. Also, the use of non-coloured solutions (such as quinine or non-coloured tonic water) prevents the operator is influenced by the native colour of the marker, as for example, would occur with fluoresceine (a yellow-orange solution), during the activity, promoting a more immersive and realistic environment.^10^ In the present study, the dilution of the original solution was not performed because tonic water was transferred into an empty IV bag (a procedure identical to that performed in the handling of cytotoxic drugs not subject to dilution prior to IV administration, such as doxorubicin and nab-paclitaxel). Still, all the performed procedures are identical in both cases and there is no increased/diminished risk of spillage biasing the results. In line with the procedures usually performed for the preparation of cytotoxic drugs and with similar previously published studies, the present study included the aspiration of solutions from vials, the connection of the aspiration syringe to the liquid transfer system and the addition of the test solution to the perfusion bag.^10,11,14^

**Figure 2.**
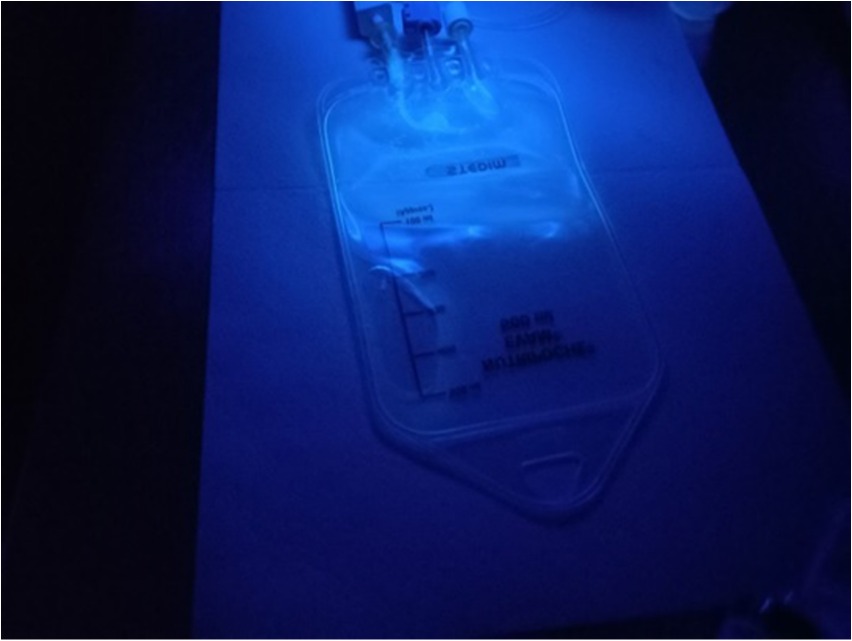
Positive control obtained by applying ultraviolet (UV) light in an intravenous fluid administration bag containing tonic water.

The application of the tonic water fluorescence tests to 12 volunteers with prior experience on cytotoxic drugs manipulation, corroborated the adequacy of the test since spillages were clearly identified under UV light. The display of fluorescence by the infusion bag, after transfer of tonic water, removes any doubt as to the suitability of the solution as a fluorescent marker and can be considered a positive control (Figure 2).

Seven of the participants (58%) were found to have spills resulting from their handling procedures, on 11 of the 288 total surfaces assessed (4%) (Table 2). These results are similar to those previously published by Baussant et al.,^11^ in which eight of the ten professionals evaluated reported spills resulting from handling acts.

**Table 2.** Percentages of spillages resulting from the handling of tonic water carried out by pharmacy technicians with prior experience in handling cytotoxic drugs (*n* = 12).

Analysed criterion	Percentage (%) (Cases / Total of opportunities)
Professionals with identified spills	58% (7/12)
Contaminated surfaces	4% (11/288)
Professionals with spills identified in needle manipulation	42% (5/12)
Professionals with spills identified in spike manipulation	17% (2/12)
Surfaces contaminated in needle manipulation	6% (9/144)
Surfaces contaminated in spike manipulation	1% (2/144)
Working field among the total of contaminated surfaces/materials/equipment	36% (4/11)
Gauze dressings among the total of contaminated surfaces/materials/equipment	36% (4/11)
Vials among the total of contaminated surfaces/materials/equipment	27% (3/11)

**Figure 3.**
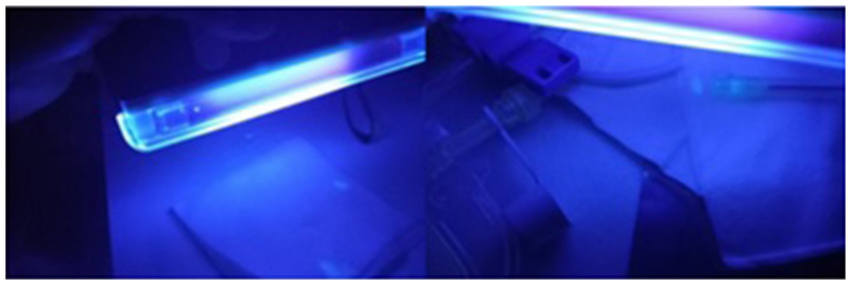
Detection of tonic water spills under ultraviolet (UV) light during manipulation simulation.

Needle manipulation is considered more dangerous due to the higher probability of aerosol release (not assessed in this study), greater difficulty in managing the pressure inside the vial, and the risk of puncture for the operator.^15^ In the present study, manipulation with needle also resulted in a higher number of spills than manipulation using a pressure release system spike. These results suggest the need to avoid, whenever possible, the use of needles in the manipulation of cytotoxic drugs. The materials/equipment/surfaces where spillages were observed included the tonic water vial, the working field and gauze dressings (Figure 3).

According to OSHA, a sterile gauze dressing should always be wrapped around the needles and the vial cap when withdrawing the solution.^8^ One of the practices also often associated with a higher probability of spillage is the transfer of cytotoxic drugs to empty perfusion bags, or containing diluents such as 0.9% sodium chloride or 5% glucose.^11^ Accordingly, OSHA recommends the use of gauze dressings between the luer-lock connection areas.^8^ Placing dressings between the operator's glove and connection zones (needle/spike and vial; syringe and fluid administration system) was invariably complied with by the participants and proved very important to avoid the transfer of tonic water residues onto the operators’ gloves. The use of adequate working fields to absorb cytotoxic spills is also important to limit their passage, deposition and accumulation in the manipulation area.

**Figure 4.**
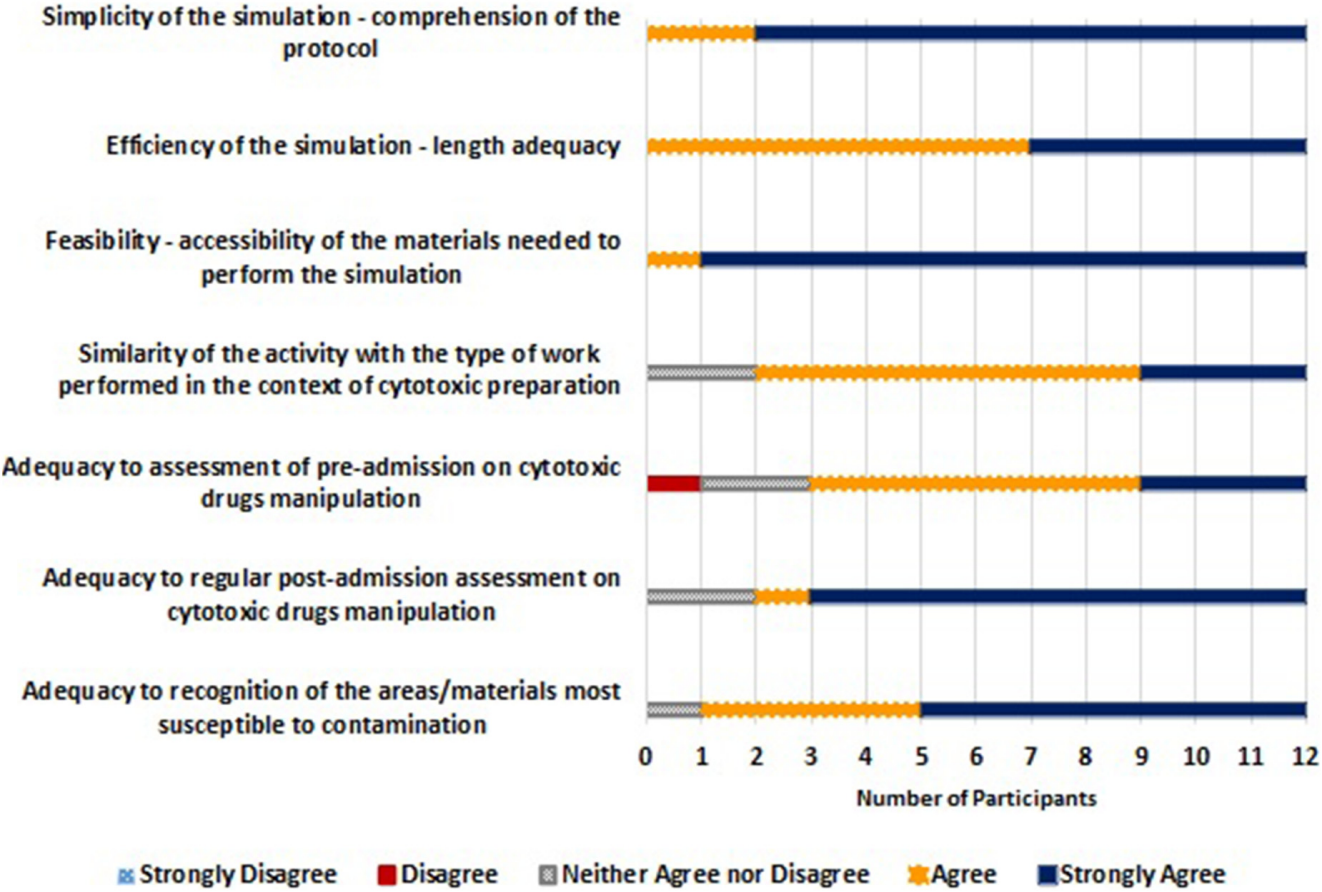
Evaluation of the suitability of the simulated manipulation protocol of tonic water by the study participants (*n* = 12).

All participants agreed or strongly agreed that the manipulation simulation with tonic water was simple, efficient and feasible. Most participants either agreed or strongly agreed that the simulated manipulation is similar to the type of work that they usually perform in the manipulation of cytotoxic drugs and is an adequate means to assess new or experienced professionals and to identify the areas/materials more prone to spillages (Figure 4).

## Conclusion

This study identified an easily accessible, inexpensive and harmless solution that may increase the adherence of professionals and services to training and assessment in simulated handling of cytotoxic agents, as universally recommended by guidelines. The simulation of manipulation with tonic water confirmed the occurrence of spills during cytotoxic drugs handling, particularly when using needles, which further reinforces the need to use alternative puncture devices. The use of gauze dressings in device connection zones also proved to be fundamental since they were among the products with the highest tonic water residues. The simulated manipulation was featured with efficiency, simplicity and feasibility, which might be particularly useful for the promotion of training and assessment in less-resourced settings.
